# Wheelchair Navigation System for Disabled and Elderly People

**DOI:** 10.3390/s16111806

**Published:** 2016-10-28

**Authors:** Eun Yi Kim

**Affiliations:** Visual Information Processing Lab., Konkuk University, Seoul 143-701, Korea; eykim@konkuk.ac.kr; Tel.: +82-2-450-4135

**Keywords:** intelligent robots, image analysis, object recognition, assistive technology, computational intelligence

## Abstract

An intelligent wheelchair (IW) system is developed in order to support safe mobility for disabled or elderly people with various impairments. The proposed IW offers two main functions: obstacle detection and avoidance, and situation recognition. First, through a combination of a vision sensor and eight ultrasonic ones, it detects diverse obstacles and produces occupancy grid maps (OGMs) that describe environmental information, including the positions and sizes of obstacles, which is then given to the learning-based algorithm. By learning the common patterns among OGMs assigned to the same directions, the IW can automatically find paths to prevent collisions with obstacles. Second, it distinguishes a situation whereby the user is standing on a sidewalk, traffic intersection, or roadway through analyzing the texture and shape of the images, which aids in preventing any accidents that would result in fatal injuries to the user, such as collisions with vehicles. From the experiments that were performed in various environments, we can prove the following: (1) the proposed system can recognize different types of outdoor places with 98.3% accuracy; and (2) it can produce paths that avoid obstacles with 92.0% accuracy.

## 1. Introduction

With increases in the number of elderly and disabled people, there is growing demand for support devices and care equipment to improve their quality of life. Over the last 20 years, the electric powered wheelchair (EPW) has been the most common mobility aid for those with reduced mobility and, recently, the intelligent EPW, which is also referred to as an intelligent wheelchair (IW), has garnered considerable attention as an emerging device to meet users’ various demands [1,2,3,4,5,6,7,8]. Essentially, IWs are EPWs with an embedded computer and sensors, which gives them “intelligence”. The important evaluation factors of such IWs are safety and ease of operation; thus, two basic techniques should be developed for IWs: (1) convenient interactions between the users and the IWs, which allow disabled users to manipulate the wheelchair themselves using their own physical abilities; (2) a navigation service that offers obstacle detection, recognition of surrounding environments and collision avoidance.

In previous work [3], a vision-based interface for the severely disabled was developed for control of the IW, and its effectiveness was demonstrated via experiments and field tests. Consequently, this paper will discuss the navigation techniques for IWs that allow users with both cognitive and motor impairments to have safer mobility.

During the past decade, a number of navigation algorithms have been explored for IWs, and most of them have used various range sensors for obstacle detection and avoidance [4,5,6,7,8,9,10,11,12]. These sensor-based navigation systems consider objects that protrude more than a given distance from the ground as obstacles. The NavChair was developed for elderly people [4] and can recognize various obstacles using infrared (IR) sensors and ultrasonic ones, thereby assisting the user in navigating narrow spaces. The robotic wheelchair was designed in [5] and offers obstacle avoidance and automatic following of target places specified by a user. In addition, a drive safe system (DSS) was implemented to support the safe mobility to visually impaired users [6,7]. The DSS can identify obstacles using two bumpers, five IRs, and five ultrasonic sensors, and it allows the user to follow a wall and cross through doors inside a building. Because such sensor-based navigation systems are simple and easy to install, they have been widely used for obstacle avoidance systems in mobile robots and manipulators. However, they are hampered by specula reflections and a poor angular resolution. Moreover, to recognize a variety of obstacles such as flat-shaped or small-sized objects, they require a number of sensors with higher capacities.

Vision-based navigation has received intensive attention as an alternative solution to sensor-based navigation [13,14,15,16,17,18,19,20,21], and these methods are further categorized into stereovision-based methods and monocular vision-based ones. The methods using stereovision techniques discern obstacles using three-dimensional depth information. The significant drawback of these methods is that they involve high computing time and hardware costs. In contrast, monocular vision-based navigation systems use image processing and computer vision techniques to perceive obstacles, regarding all objects different to the ground as obstacles. Accordingly, an appearance model that describes the visual characteristics of the background is first identified, and it must be robust to some environmental factors, such as background complexity and illumination. The study in [20] proposed a background estimation model that can be learned easily in real time and that works well on both textured and textureless backgrounds; the online model also improves the system’s sensitivity to the type of illumination.

Although many navigation systems work well both indoors and outdoors, some significant problems remain. An important issue is that such navigation systems’ performance is too sensitive to noise and can overlook some obstacles. In the literature, numerous obstacle avoidance methods have been proposed and applied to the intelligent wheelchair, most using the potential fields approach (PFA), vector field histogram (VFH) or dynamic window approach (DWA). The PFA was first suggested in [22], and developed later by Khatib who used the PFA as a real-time obstacle avoidance approach for mobile robots [23]. Recently, it has been also used for collision avoidance with the IW [24,25]. The concept combines positive forces acting at the goal with negative forces emerging from obstacles; it then builds a gradient descent trajectory from the current position to a specific target, which is well suited to real-time mobile robot navigation. One major problem occurring when using the application of PFA is the local minima problem, which can occur, for example, in the case of passage between two close obstacles such as doorways. Recently, some methods have been proposed to resolve this problem [26]. The VFH was proposed in [27], as an obstacle avoidance for fast robots, which is performed as follows: First, it produces 2D/3D occupancy maps and then transforms them to 1D polar histograms; then, it determines the sectors that have an obstacle density below the threshold for candidate directions and navigates the wheelchair. The VFH performance depends greatly on the results of the obstacle detection. Therefore, an error in the obstacle detection can directly result in collisions with obstacles and wheelchairs. The DWA is well-suited for high-speed operating robots in [28], and has been recently used for the wheelchair on its own or in combination with another algorithm. For example, a hybrid shared control method that combines the merits of the combined VFH and the DWA was used in [29].

Another important issue to consider in wheelchair navigation is the discrimination of traffic intersections with other situations, such as sidewalks and roadways. Most accidents in real outdoor environments have occurred at traffic intersections. As such, these places are more dangerous for people with physical or cognitive disabilities because misclassification can lead the catastrophic accidents that could result in fatal injuries to the user [30]. For full safety, a mechanism that recognizes outdoor situations must also be integrated into IWs.

In this work, a novel IW navigation system is presented that provides more safety for people with impairments and for elderly people. In order to assure safe mobility, our IW offers the following central functions: obstacle avoidance and situation recognition. With these two functions, it can perceive obstacles of various types and recognize dangerous situations, and then recommend viable paths to evade them. First, obstacles are identified using a combination of one vision sensor and eight ultrasonic ones, and then the avoidable directions are determined using learning-based algorithms. Second, in order to prevent collisions with vehicles at traffic intersections, the situation recognition component distinguishes the type of place where the user is currently located as a sidewalk, an intersection or a roadway according to the texture and shape characteristics.

The primary contribution of the proposed method is two-fold. First, it can guarantee greater safety to disabled and elderly people through recognizing obstacles and outdoor situations. Second, it can determine more accurate viable paths in various environments using the learning-based filters to automatically find important visual clues despite overlooking some obstacles or errors in detecting some obstacles.

In order to evaluate the usefulness of the proposed IW, several datasets were collected from real environments with various types of illumination and complex structures, and then experiments were conducted. The results were compared with the ground truths collected by human vision. Then, the experimental results demonstrated that our navigation algorithms can recognize different types of outdoor places with 98.3% accuracy and can produce avoidable paths with 92.0% accuracy in indoor and outdoor environments. Furthermore, in order to show its practicality, a field test was undertaken with five disabled users, which demonstrated that all participants could fulfill the navigation mission along the tracks with relative safety and ease.

## 2. Our Intelligent Wheelchair (IW) System

This work aims to provide safe mobility to wheelchair users while they control the wheelchair toward a destination. In order to support safe mobility, the wheelchair must detect a range of obstacles and dangerous situations in real environments and generate avoidable paths to prevent collisions with them. In order to achieve this, a hybrid obstacle avoidance method and a situation recognition method are proposed.

**Figure 1:**
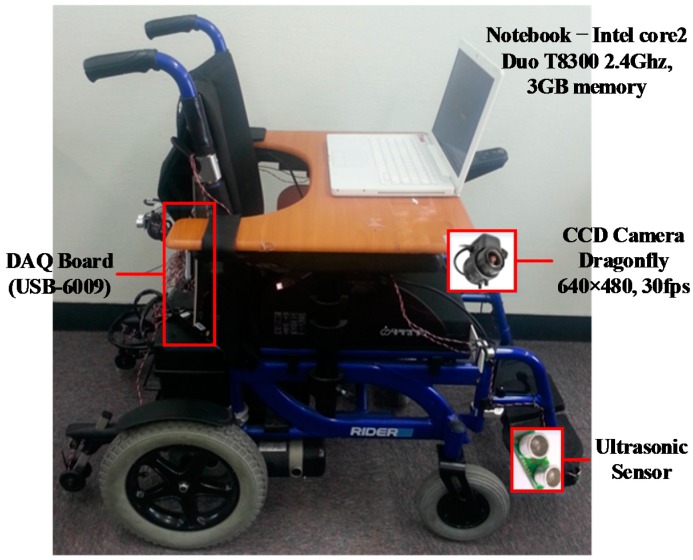
Overall architecture of the proposed IW.

Figure 1 depicts the architecture of the proposed IW system, which consists of a general EPW, a charge-coupled device (CCD) camera, eight ultrasonic sensors, a notebook, and a data acquisition (DAQ) board. By processing the image streaming acquired from the CCD camera, the user can recognize upcoming obstacles and the type of place, thus preventing collisions with diverse obstacles, including walls, pedestrians, and moving vehicles at traffic intersections.

**Figure 2:**
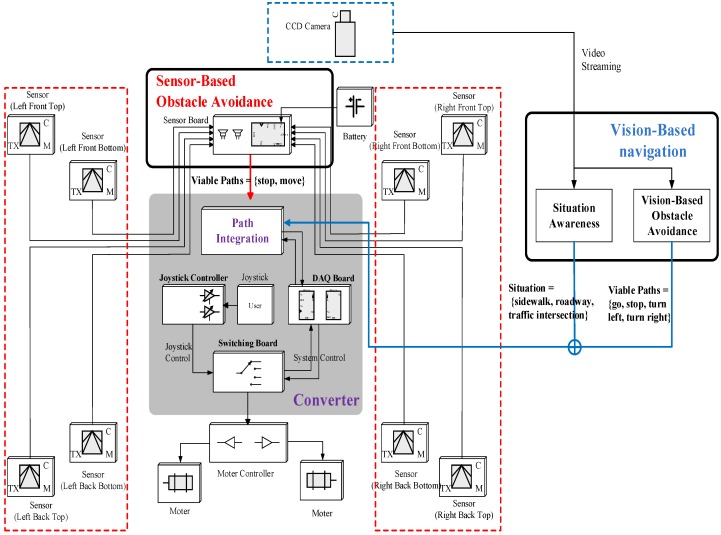
Data flowchart of the proposed intelligent wheelchair (IW).

Figure 2 presents the flowchart that demonstrates how the sensor values are processed and conveyed to the user (or wheelchair). The proposed IW is made up of four modules: sensor-based obstacle avoidance, vision-based obstacle avoidance, situation recognition, and converter. While a wheelchair user is moving, the proposed wheelchair system detects diverse obstacles and determines a path to evade them. In order to achieve this, a hybrid navigation system is employed using both range sensors and a CCD camera. Most obstacles and dangerous situations are detected using vision-based navigation: obstacles of various types are discriminated using a background color model that is continuously updated via online learning, and the viable paths used to avoid collisions with the detected obstacles are determined using the learning-based classifiers. Furthermore, in order to prevent collisions with vehicles at traffic intersections, the situation recognition module determines if the user’s location is at an intersection or on a sidewalk. Then, in order to process a blind area that cannot be covered by the CCD camera, the IW uses eight range sensors that are attached to the foot tray and the corners of the IW. The processed results are conveyed to the converter, which decides the most suitable paths, and delivers it to the user or directly controls the wheelchair.

## 3. Situation Recognition

The most dangerous situation to a pedestrian, particularly wheelchair users, is a traffic intersection, because collisions with vehicles can occur that result in fatal injuries. Thus, a technique to recognize the current situation of a user is essential. In this work, situations are categorized as sidewalks, traffic intersections, or roadways. In order to identify outdoor situations, texture classification and shape filtering were successively conducted on the image streaming obtained from a camera sensor.

### 3.1. Texture Classification

Firstly, the smoothing using a Gaussian filter and the contrast correction using histogram equalization are successively applied to the image. Then, the input image with a size of 640 × 480 pixels is divided into 768 blocks with sizes of 20 × 20 pixels, and the texture classification is conducted on the blocks. In order to discern the boundaries between the roadways and sidewalks from other natural contours, the texture characteristics of the blocks were explored. In this work, in order to capture the diversity in a texture pattern, both colors and histogram of oriented gradient (HOG) are used. The HOG is a descriptor that counts the frequencies of an edge orientation in the blocks and is primarily employed for object detection [21]. The HOGs are computed for all 20 × 20 sized blocks, as follows: (1) we compute gradient magnitude and orientation of each pixel using the Sobel operator; (2) we discretize each pixel’s gradient orientation into six histogram bins, and accumulate the pixels’ magnitudes to the corresponding bins of the HOG; (3) we normalize the HOG by the area of a sub-region. Therefore, ${\mathrm{HOG}}_{R}$ is defined as

(1) $$HOG_{B}=\left\{HOG_{B}\left(j\right)=\frac{1}{Area\left(B\right)}\times \underset{k\in B}{\sum }mag.\left(k\right),if\;gradient\left(k\right)=j,\;1\leq j\leq 6\right\}.$$

In (1), the Area(B) is the number of pixels belonging to a block *B*, and the mag.(k) and the gradient(k) are the gradient magnitude and the orientation of the pixel k, respectively.

In this work, we assume the pixels belonging to roadways have a distinguishing saturation distribution. Thus, we employ the mean of the pixels’ saturation within a block ($S_{B}$), to demonstrate the color information.

According to such textural characteristics, a rule-based decision is conducted on every block. A block is regarded as a boundary class if both of the following conditions are satisfied: the ${HOG}_{B}$ has a larger variance than a predefined threshold ($\theta_{H}$) and $S_{B}$ is smaller than the threshold $\theta_{S}$. Through experiments, the two values were fixed at 2 < $\theta_{H}$ < 20 and $\theta_{S}$ < 20. Accordingly, a binary image is generated after texture classification.

### 3.2. Shape Filtering

In this stage, the type of outdoor location is determined according to the boundaries’ orientations: if they are horizontally aligned, the image is regarded as an intersection; if they are aligned close to vertical, the image is regarded as a sidewalk. In order to reduce the effects of misclassified blocks and to conclude the correct situation, a profile analysis was conducted on the binary images.

A binary image is projected along the x-coordinate and the y-coordinate, and two histograms are calculated: a horizontal histogram and a vertical histogram. Then, the following three rules are applied sequentially in order to classify the current situation: (1) An intersection is indexed to the image where some values on the horizontal histogram are above the threshold; (2) An intersection is indexed to the input image where the values on a vertical histogram have a uniform distribution; (3) A sidewalk is indexed where the variance of vertical histogram is larger than the threshold *σ*. Here, 10 was set to the threshold through experiments.

**Figure 3:**
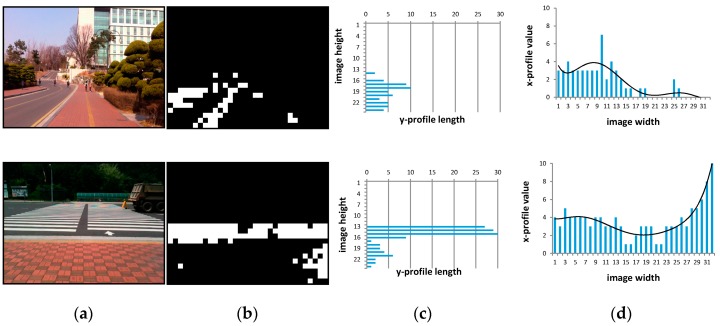
The examples of shape filtering results: (**a**) input images; (**b**) binary images after texture classification; (**c**) horizontally projected profiles; and (**d**) vertically projected profiles.

Figure 3 depicts the process of situation recognition. Figure 3a,b show the input image and the texture classification result, respectively. As shown in Figure 3b, most blocks are correctly classified into boundary and non-boundary classes. According to the classification results, the current situation is determined. Figure 3c,d are the *y*-axis projection profile of a binarized image and the *x*-axis projection profile, respectively. As seen in Figure 3c, the first-row image has a vertical histogram with a larger variance; therefore, its situational class is regarded as a sidewalk. In contrast, in the second-row image, some values on the horizontal histogram are larger than the threshold, so its situational class is understood to be an intersection.

## 4. Obstacle Avoidance

In the proposed system, in order to recognize a variety of obstacles including static obstacles and moving obstacles, both the vision sensor and eight ultrasonic ones are used. The eight ultrasonics can only measure obstacles that are placed within 3 cm to 2 m from the wheelchair. In contrast, the camera can detect obstacles that are positioned from 0.4 m to 14 m. Thus, most obstacles are identified using the vision-based obstacle avoidance algorithm, and only stairs and obstacles coming from behind the wheelchair are detected by the sensor-based one.

### 4.1. Vision-Based Obstacle Avoidance

#### 4.1.1. Generation of Occupancy Grid Map

An occupancy grid map (OGM) demonstrates the environmental information, such as the position of an obstacle and its size, each cell of which represents the risk of the corresponding area by gray color values. In this work, we used a camera that has a focal length of 120°, which is comparable to human vision, and that has a resolution sized at 320 × 240 pixels. In this stage, the image is transferred to a 32 × 24 OGM via an online background model estimation and a background subtraction.

In this study, the background model is obtained using the simple learning algorithm developed in [21]. The background model is calculated from the reference area only, that is, a 1 m trapezoidal area in front of the camera. The input image is first smoothed using 5 × 5 Gaussian filters and then its color space is transformed to the Hue-Saturation-Intensity (HSI) one. For the pre-defined reference area, two histograms are calculated for the hue and intensity, which are denoted as $BH_{t}$ and $BH_{t}$, respectively. These histograms are accumulated for the five most recent images, which are employed for computing the model of background. Thereafter, such a background model is continuously updated with each new frame input.

Once the background model is determined, the image subtraction is conducted with an input image and a background model, as follows:

(2) $$\{\begin{array}{c} M_{t}=1\;if\;\left(\left|BH_{t}\left(s\right)-H_{t}\left(s\right)\right|\right)\leq T_{H}\;and\;\left(\left|BI_{t}\left(s\right)-I_{t}\left(s\right)\right|\right)<T_{I}\\ M_{t}=0\;otherwise \end{array},$$

where $T_{I}$ and $T_{H}$ are the thresholds for the $BI_{t}$ and the $BH_{t}$, respectively. In this study, the thresholds are set to 80 and 60, respectively. A pixel with a smaller difference than the threshold is regarded as belonging to the background, and a pixel with a larger difference is regarded as the obstacle. Accordingly, a binary image is generated for every frame.

According to the background subtraction results, the OGM is generated, each cell corresponding to one block of 10 × 10 pixels in the binary image, and its color representing the risk level of the corresponding area. Here, ten scales are employed according to the level of risk. Accordingly, the gray color of a cell is determined as follows: 0.1 × (number of pixels classified as background). The more intense a grid cell, the more closely spaced the obstacles.

#### 4.1.2. Viable Path Recommendation

Despite the online learning-based background estimation, some misclassification can happen due to the time-varying illumination. In order to correct the effect of such lightening conditions, a learning-based path recommendation is presented that can automatically catch the common patterns among the OGMs that are assigned in the same directions. In the proposed IW, the wheelchair is controlled according to four directions—“go straight”, “stop”, “turn left”, and “turn right”. Here, we employ two machine learning algorithms and finally select the algorithm that has the better accuracy. First, a neural network (NN) is used as a classifier, which consists of 768 input nodes, 110 hidden nodes, and four output nodes. It uses gray values on a 32 × 24 sized OGM as inputs, then outputs four floating numbers, each of which describes the possibilities of the respective four directions to be chosen as the avoidable path. Back-propagation was used for the NN learning. In the online test, the direction with the highest output among the four directions is determined to be the avoidable one. Unlike the NN, which allows for multi-class classifications, the standard support vector machine (SVM) is applied to binary classification problems. In order to directly apply an SVM to a four-direction classification, a one-vs-all scheme is used. The decision is performed in three steps: the first classification is performed to categorize the current situation into “move” or “stop”; the second classification of “go straight” and “turn” is determined; and the third classifier is used for the classification of “turn left” and “turn right”. The SVMs receive the same feature vectors to the NN as an input, and they use a linear kernel with *c* = 10.

Two classifiers were tested with numerous images collected from real indoor and outdoor environments, and the results demonstrated that the SVM-based classifier performed better than the NN-based classifier, which is discussed in detail in the experiments (in Section 6.1).

**Figure 4:**
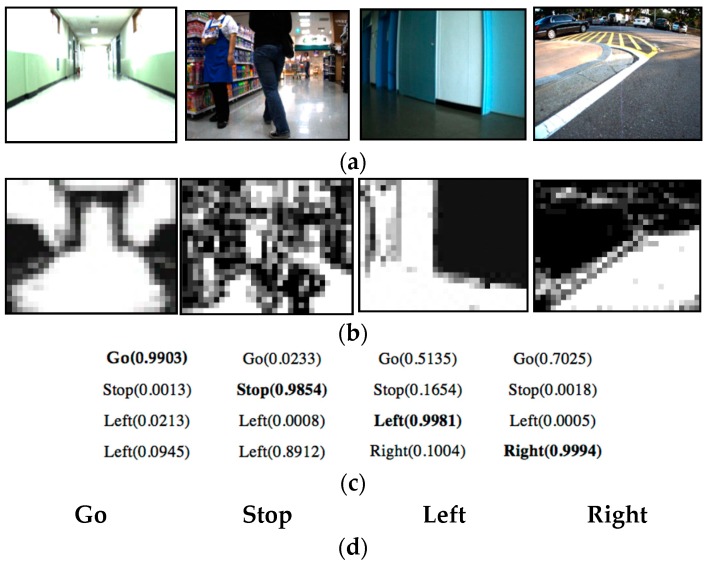
Some results of OGM generation and path recommendation results: (**a**) input images; (**b**) OGMs; (**c**) recognition results when using the NN-based classifier; and (**d**) recognition results when using the SVM-based classifier.

Figure 4 presents the results of the vision-based obstacle avoidance algorithm. Figure 4a,b show the input image and the generated OGMs sized at 32 × 24. As seen in these images, the learning-based method recommended accurate viable paths.

### 4.2. Sensor-Based Obstacle Avoidance

In the proposed system, SRF005 ultrasonic range sensor is used to detect objects, which is sensitive enough to detect a 1 cm diameter broom handle at a distance of over 2.4 m. Although attaching a number of sensors guarantees more safety, it involves a higher computation cost to process the sensor values. Thus, in this study, only eight sensors were attached to the IW, in order to handle areas that cannot be covered by the CCD camera.

**Figure 5:**
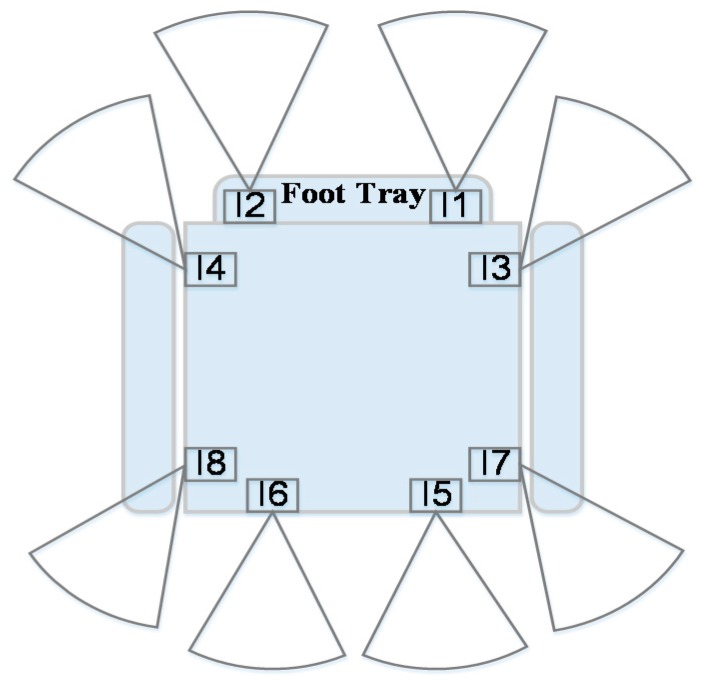
Sensor positions attached to our IW system.

Figure 5 describes the positions of the sensors attached to the proposed IW system, all of which have a maximum range of 2 m and a minimum range of 1.03 cm. Through processing the information measured from sensors, the method categorizes the current paths into “move” and “stop”, and it is primarily employed to recognize stairs and obstacles coming from behind the wheelchair.

Sensors I1 and I2, which are attached to the foot tray of the wheelchair, are used to detect stairs. If the distances measured by the sensors increase, it is assumed that the user is going to approach downward stairs or a cliff, thereby making the user stop. The two sensors I3 and I4 are used to detect obstacles that approach abruptly from both sides of the wheelchair. Finally, sensors from I5 to I8 are used to detect obstacles coming from behind the wheelchair: if their distances decrease, the wheelchair user stops. The wheelchair commands based on these operations have higher priorities than commands generated by the vision-based obstacle avoidance in order to ensure safety.

## 5. Converter

The primary role of this module is to deliver the recognition results to the user. In order to achieve this, it first integrates all recognized results from the three modules—situation recognition, vision-based obstacle avoidance and sensor-based obstacle avoidance—and decides more suitable paths. Thereafter, it conveys the decision to the user.

### 5.1. Integration of the Recognition Results

All recognized results from the situation recognition and the obstacle avoidance methods are delivered to the converter, and then the more suitable paths are determined to support safe mobility for the user. The decision function for finding the best paths among the results received from the three modules is illustrated in Table 1.

### 5.2. Wheelchair Control

The selected viable path is conveyed to the user. In this work, two different control schemes are provided: joystick control and system control. This is determined in the converter.

**Figure 6:**
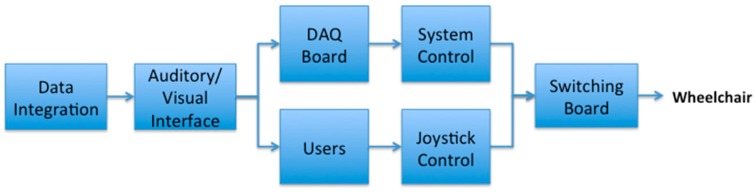
Wheelchair control mechanism according to the user types.

As seen in Figure 2, the converter is composed of a DAQ board, a joystick controller, and a switching board. Through the switching board, the user can select the control type, which is determined by the user’s need, e.g., disability, age, etc. If the user has a severe cognitive impairment or motor impairment, the system control is chosen; otherwise, the joystick control is adopted in order for the user to directly control the wheelchair while taking the paths suggested by the navigation system. This mechanism is illustrated in Figure 6.

**Figure 7:**
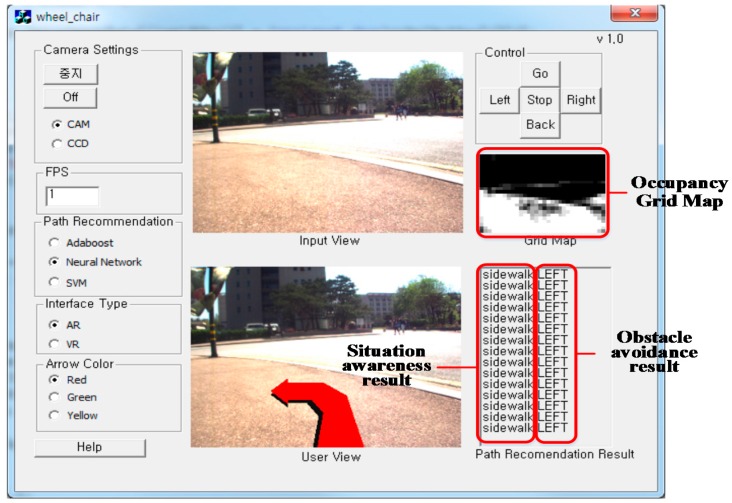
Graphical user interface.

In order to aid user understanding, a graphical user interface is provided and is depicted in Figure 7. With the proposed interface, the user can more efficiently control the wheelchair. All recognition results, i.e., the viable paths and current situations, are displayed on the laptop computer’s screen. In particular, the viable path is overlaid on the real-time image, so the user can direct the wheelchair along the viable path more effectively.

In contrast, some disabled people have difficulties in controlling the wheelchair in a timely manner, so they can collide with some obstacles. In order to prevent these situations, we allow the system to directly control the IW.

As depicted in Figure 6, the proposed IW uses a DAQ board to change the recognized results to wheelchair control commands. Similar to the EPW that is manipulated according to the voltage, a DAQ board (USB-6009) is used to transfer the digital commands to analog commands. The board is connected to the notebook and it is programmed using Visual Basic. Through the DAQ-board program, the directions of the wheelchair are directly controlled by simply modifying the voltage passing via the wheelchair.

Table 2 describes the command map between the wheelchair directions and output voltages to the two motors. By producing different output voltages, the proposed system can control both the direction and velocity of the wheelchair.

## 6. Experimental Results

To evaluate the validity of the proposed navigation method, experiments were conducted on a number of images collected from both indoor and outdoor environments. For its practical usage as mobility aids, the device has to be robust to various situational factors, such as place types and lighting conditions. Therefore, 80,000 images from the indoors and outdoors were collected over a period of one year. Thereafter, the ground truths for all images were annotated by humans. The images were used to evaluate our outdoor situation recognition method and our obstacle avoidance method.

This section is composed of three sub-sections: Section 6.1 depicts the results for the proposed obstacle avoidance method and Section 6.2 demonstrates the results for situation recognition method. Finally Section 6.3 reports the navigation results by the proposed wheelchair control mechanism.

### 6.1. Obstacle Avoidance Results

For evaluating the performance of the obstacle detection and obstacle avoidance, a large amount data is investigated.

Table 3 presents the dataset used to evaluate the obstacle avoidance method. A total of 80,000 images were divided into six datasets according to their illumination types, background types, and obstacle types. From the datasets, the training data was randomly selected, then only 1000 images were used to train learning-based classifiers such as the SVM and NN.

**Figure 8:**
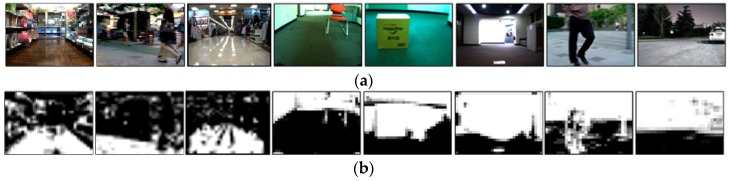
Some examples of obstacle detection: (**a**) input images and (**b**) the OGMs.

Figure 8 presents the results for detecting obstacles under various environments, where the first to sixth columns are the detection results for indoor static obstacles with various lighting conditions, and the last two columns are the results for outdoor moving obstacles. In more detail, the first three columns present the classification results for complex lighting conditions, and the fourth column describes the detection results for a thin static obstacle that is floating. The fifth column is the result for detecting a thick static obstacle. In contrast, the sixth column presents the detection of a small thin obstacle at night. Finally, the seventh and eighth columns describe the results for detecting outdoor moving obstacles during the daytime and the nighttime, respectively. For the given input images in Figure 8a, the generated OGMs are presented in Figure 8b. As you can see in the figure, the proposed method can correctly discriminate a variety of obstacles, from flat-structured obstacles to large-sized obstacles such as people and cars. Furthermore, the proposed method with a single camera can process obstacle classification in real time using a simple online background learning scheme.

After obstacle detection, the viable paths need to be determined. In order to achieve this, a learning-based path recommendation is proposed. Here, two classifiers, an NN and an SVM, were employed. Thus, a performance comparison between the two classifiers was undertaken.

In addition to showing the effectiveness of the proposed method, the performance comparisons with an existing method using the VFH [27] were performed. The method first produces 2D/3D occupancy maps and transforms them to 1D polar histograms; then, it determines the sectors that have an obstacle density below the threshold for the candidate directions and controls the wheelchair.

**Figure 9:**
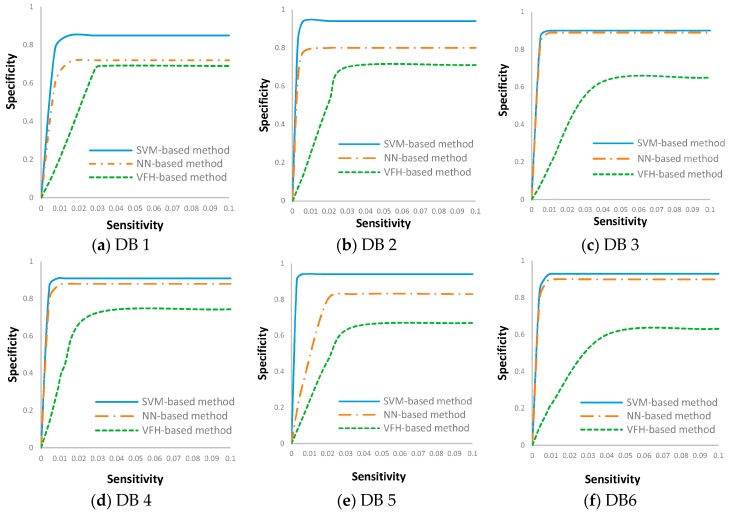
Accuracies of three obstacle avoidance methods: (**a**–**c**) are results for indoor datasets and (**d**–**f**) are ones for outdoor datasets.

Figure 9 depicts the performance summary of the three obstacle avoidance methods, i.e., the VFH-based method, NN-based method, and SVM-based method, which is analyzed using the receiver operation characteristic (ROC) curve. The ROC curve can provide a more detailed comparison of the three methods. It is drawn with two axes: sensitivity and specificity. The sensitivity represents the partition of the actual positives that are correctly identified as such. In contrast, the specificity represents the ratio of negatives that are correctly identified as such. That is,

$$sensitivity=\;\frac{number\;of\;true\;positives}{number\;of\;true\;positives+number\;of\;false\;negatives}$$

and

$$specificity=\frac{number\;of\;true\;negatives}{number\;of\;true\;negatives+number\;of\;false\;positives}$$

As depicted in Figure 9, the ROC curves clearly exhibited differences among the three methods. Among the three methods, the SVM-based method exhibited the fastest convergence speed as well as the highest accuracy. Although there were some differences according to the environments, the SVM-based classifier converged to approximately 99.5% sensitivity and 90.0% specificity regardless of the environments, and the NN-based method converged to almost 98.4% sensitivity and 90.0% specificity. In contrast, the VFH-based method gradually converged to 96.0% sensitivity and a specificity between 60.0% and 70.0%.

Table 4 describes the average performance of the three methods for indoor and outdoor environments.

From these results, the following conclusions can be deduced. (1) Regardless of the environment, the proposed obstacle avoidance method exhibits superior performance compared with the VFH-based method that has been often used in the literature. On average, the SVM-based classifier can recommend viable paths with an accuracy of 88.0% and 92.0% in indoor and outdoor situations, and the NN-based classifier has an accuracy of 83.8% and 89.0% in the respective environments. In contrast, the VFH-based method has an accuracy of 68.0% and 68.8%, respectively; (2) Between the SVM-based and NN-based classifiers, the former exhibited better performance than the latter. Furthermore, the SVM-based method was more robust to lighting conditions and ground types than the NN-based method.

In order to be practically used as mobility aids, real-time processing should be guaranteed in the proposed IW. Thus, a comparison of the processing times was also conducted. Table 5 describes the processing time for the respective methods. For the implementation of the three classifiers, the NN-based method was the fastest, followed by the SVM-based method, and finally the VFH-based method. Even though the SVM-based method involved more computational cost than the NN-based method, its accuracy was the highest, so the SVM-based method was adopted for implementation in the IWs.

As described in Table 5 and Figure 9, the numerical comparisons demonstrated that our obstacle avoidance method is more accurate in detecting a diversity of obstacles and determining viable paths. Moreover, the average time taken for the proposed method to process one frame was approximately 5 ms, thereby allowing real-time processing. The proposed method was approximately 290 ms faster than the VFH-based method. Consequently, the proposed method can improve the recognition of potential collisions and the recommendation of viable paths compared with the existing methods, thereby providing a wheelchair with safe navigation in real environments.

### 6.2. Situation Recognition Results

A total of 1742 images were used for evaluation of the proposed situation recognition method, which were divided into six datasets in accordance with their environmental factors. Table 6 lists the datasets used in our experiments.

Out of 1742 images, 174 images were employed for determining the optimal parameter set ($\theta_{H}$, $\theta_{S}$, σ), which was employed as the threshold for the stages of texture classification and shape filtering. The remaining images were employed for evaluating the proposed method.

**Figure 10:**
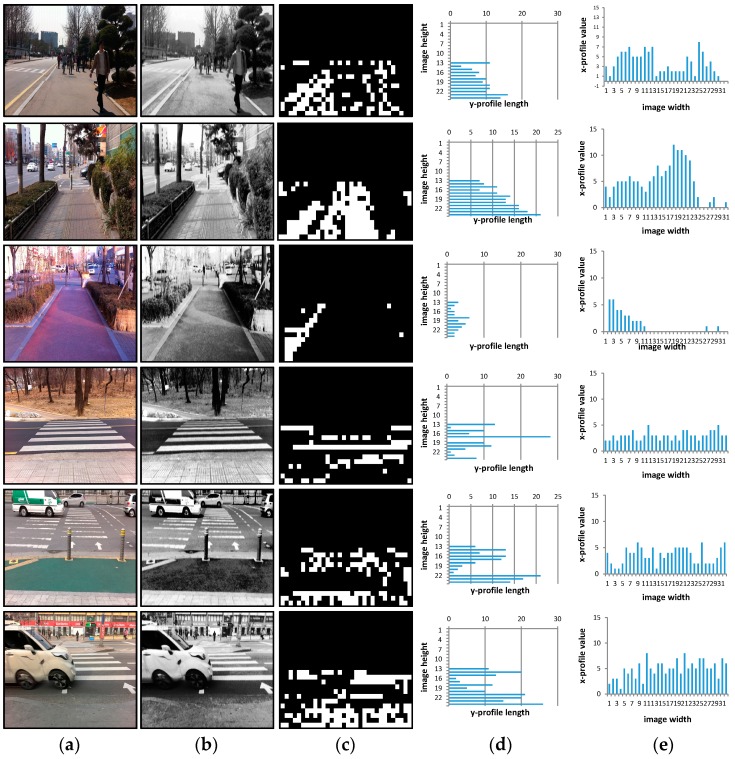
Examples of situation recognition results: (**a**) input images; (**b**) images after pre-processing; (**c**) binary images after texture classification; (**d**) horizontal histogram; and (**e**) vertical histogram.

Figure 10 presents some recognized results, where the experiments were conducted outdoors with various situations. Figure 10a depicts the input images, which have different lighting conditions and the sidewalks have various colors and patterns. Such images were first enhanced via a pre-processing; results are presented in Figure 10b. Thereafter, the texture classification and shape filtering were successively applied to the images. As seen in Figure 10c, the boundaries between the sidewalks and roadways were correctly captured despite the diverse patterns, but they still had some false identifications. To remove the effects of the misclassified blocks and to determine the correct situation, profile analyses were conducted on the binary images; horizontal and vertical histograms are depicted in Figure 10d,e, respectively. Three images from the top are categorized as sidewalks using the third rule, the next two images are categorized as intersections using the second rule, and the last image is categorized as an intersection using the first rule. The results demonstrated that our situation recognition method has robust performance for ground patterns and lighting conditions.

Table 7 shows the overall performance of the situation recognition method in several outdoor environments. On average, the accuracy was approximately 96.4%.

Table 8 describes the average processing time taken for the situation recognition module, which was approximately 228.54 min.

In the proposed system, the time taken to operate one ultrasonic is 20 min to 50 min according to the distance to the target objects from the wheelchair. When equipped with a total of eight sensors, it requires 0.2 s to 0.4 s for detecting obstacles around the wheelchair at a time.

However, because processing the sensor values is performed separately on the sensor board, it does not affect the total processing time. Accordingly, only the times taken to process the images were regarded. Then, the processing time taken for both situation recognition and obstacle avoidance was approximately 233 ms; thus, the proposed method can process more than four frames per second on a computer despite its low computational capacity. This demonstrates the potential for the proposed IW to be practically used as a mobility aid for the disabled people and the elderly one.

The experiments demonstrated that our method produced superior performance for situation recognition and path recommendation, thereby supporting safe mobility in real time for people with diverse impairments.

### 6.3. Wheelchair Control Results

The proposed IW supports the different wheelchair control schemes according to the user’s disability level: if the user has a severe cognitive impairment or motor impairment, the system control that provides fully autonomous navigation is chosen; otherwise, the joystick control is adopted so that the user directly controls the wheelchair while taking the paths suggested by our navigation algorithm.

Here, various experiments were conducted for evaluating the accuracies of autonomous IW navigation by the system control. Then, in order to prevent some accidents that result in injuries to the wheelchair user, the experiment was carried out in the situation by someone who does not require a wheelchair. We designed the experimental setups to test the accuracies of autonomous wheelchair control in several environments, such as narrow corridors, door passages and avoidance of obstacle. For the respective experimental setup, ten trials were administered for the wheelchair speed of 3.0 km/h, thereafter the number of collisions and completion time was measured.

**Figure 11:**
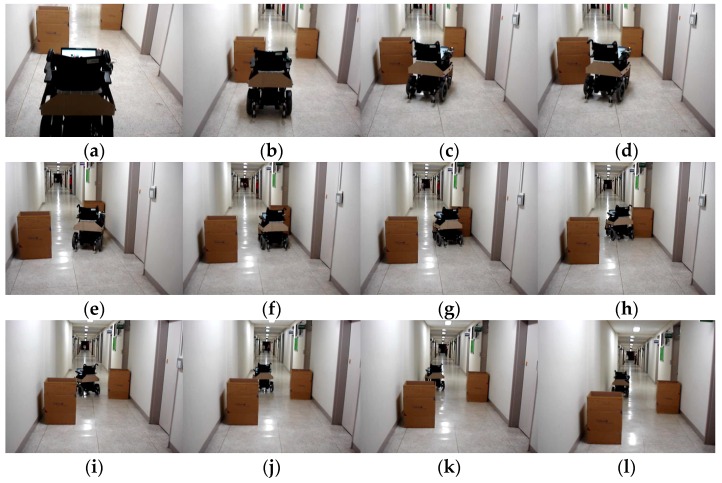
Autonomous wheelchair operation results by system control: (**a**) experimental setup; and (**b**–**l**) show the navigation results by autonomous IW control.

Figure 11a shows one experimental setup to test the IW accuracy in passing the narrow corridor while evading the collisions with two static obstacles. At the beginning, our IW was placed parallel to a wall and 3 m away from an obstacle, and driven 10 m while evading the collisions with the static obstacles. The wheelchair was driven toward the obstacles until passing through the narrow corridor. The average completion time was 15.38 ± 0.74; navigation results are shown in Figure 11b–l. As you can see in the figure, the proposed IW can successfully complete the travel without any collisions with the obstacles.

The results demonstrate that the proposed IW was capable of following a narrow corridor, passing through a doorway and avoiding some obstacles in a fully autonomous navigation mode, and that it has potential to assist the travel of disabled people with diverse disabilities. For ensuring the safety of wheelchair users, more experiments should be performed in the fully autonomous navigation mode. This issue will be addressed in our future works.

## 7. Field Test

To assess the effectiveness of the proposed navigation algorithm in the intelligent wheelchair, it was tested with five disabled users, and the results were compared with the general electric powered wheelchair.

### 7.1. Participants

Our goal is to support safe mobility to disabled or elderly people with cognitive and motor impairments. To demonstrate the validity of the proposed IW navigation algorithm, we recruited participants with different pathologies. Thus, the field test was performed on five disabled users: two men and three women, age 48 ± 25 (range 28–73 years old), who suffered from different health conditions, some with mild physical disabilities and others with severe physical disabilities. All participants were able to use a manual or powered wheelchair in their daily lives.

Table 9 provides a summary of the participants. User I is a 60-year-old male with multiple impairments, lower body amputation by diabetes and low cognition due to weak dementia. He used an EPW one year ago but now he has to rely on a caregiver to control or push it. User II is a 48-year-old female with amyotrophic lateral sclerosis. User III is a 32-year-old female with cerebral palsy. She suffers from large involuntary motion of her arms, but she can control an EPW by fine movement. User IV is a 73-year-old male with lack of strength due to weak senile dementia. His actions are very slow and he also has very poor concentration. He has used a powered wheelchair but has to rely on a caregiver. User V is a 28-year-old female with spinal cord injury, which is caused by a car accident that happened when she was 10 years old. Her actions are very slow and she has limited shoulder and hand movement due to a C3-cord injury. She has a limitation in controlling the wheelchair because of rigidity of her upper body; she can, however, use her fingers and snap them. Thus, she used our vision-based interface to control the powered wheelchair [3]. The interface uses face inclination and mouth shape information, where the direction of the IW is controlled by changing the user’s face inclination, and going and stopping are controlled by the shape of the user’s mouth. This interface was designed for severely disabled people, and its usability was demonstrated in [3]. In contrast, Users I and IV have problems in traveling independently due to cognitive impairments caused by dementia, and Users II and III could control the joystick better than the other participants.

Some ethical issues regarding this study should be mentioned: We complied with the principles and protocols of the Declaration of Helsinki when conducting the field test. First of all, to find the users to participate in our field test, we contacted the Gwanjin-gu Office (Social Welfare Division), which is a public institution in Seoul city in Korea, and explained our research goals. They approved our research, and introduced us to the Gwangjin-gu Rehabilitation Center. Thereafter, we visited the center to explain our study and allow the participants to make their own decisions. In order to give the participants insight into the research process, we gave them and their parents a short briefing about the research procedure and explained informed consent for participating in the study. After the participants indicated that they had examined the form and agreed to take part in the study, they signed the informed consent form. Throughout the field test, we repeatedly explained their right to self-determination regarding participation in our research.

### 7.2. Test Design

The goal of IW navigation is to promote users’ safe mobility and to prevent collisions with vehicles or obstacles, so the field test was designed with this goal in mind. The field test was performed in two phases, a training phase and an evaluation phase. In the training phase, we explained how to use the navigation (shown in Figure 7) interface to the participants. According to the commands suggested by the proposed method, users push the control buttons, which are shown in the top right of Figure 7. With this process, the subjects learn how to use the proposed navigation algorithm. The evaluation phase was composed of the user navigation along three established circuits and a performance analysis.

**Figure 12:**
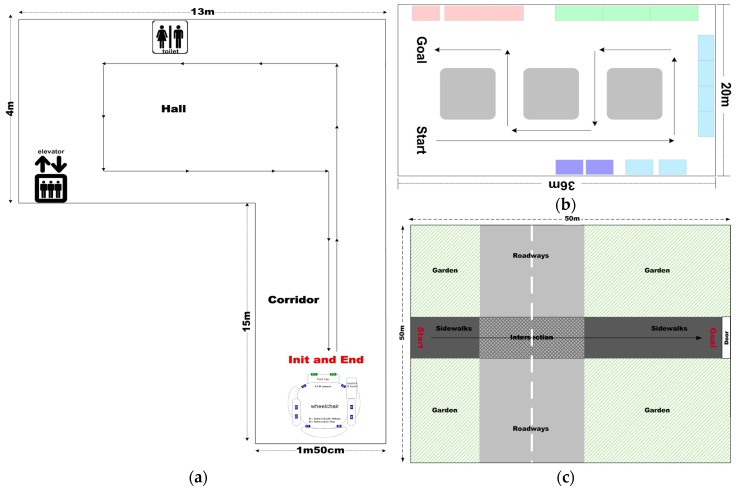
Test maps constructed for field test: (**a**,**b**) indoor maps; (**c**) an outdoor map.

In the literature, experiments were mostly performed in constrained laboratory environments, which do not accurately represent the real-life situations that people with impairments face in their daily lives. Therefore, we performed the field tests both indoors and outdoors. All the environments contained many kinds of fixed obstacles with different structural and material characteristics—chairs, fire extinguishers, narrow doorways, walls, and trees—and non-fixed obstacles (moving people or vehicles, etc.). The maps are depicted in Figure 12, which is composed of two indoor environments and one outdoor environment.

The first map, described in Figure 12a, was comprised of two spaces: a corridor and a hall. Under fixed illumination, it had a simply textured floor and only static obstacles such as walls. For the first map, the participants were asked to follow the long corridor and return to the starting point. On the other hand, the second indoor map had various obstacles—three static obstacles and one walking person—and had a more complex environment, including marble-textured flooring and mixed lighting of fluorescent and sunlight sources. For this map, we asked the participants to avoid collisions with the various obstacles, to pass narrow spaces between obstacles or between the obstacles and the walls, and to return to a pre-defined point. The route to the destination was more complex than the other maps. On the third map, the route to the destination was more dangerous due to various moving obstacles (e.g., three people walking around and two moving vehicles on the traffic intersection). The participants were asked to go straight to the predefined destination while avoiding collisions with walking people and moving vehicles. Some static obstacles, such as trees and fences, as well as people were placed on both sides of the sidewalks. The most important thing about the third map was for the users to accurately recognize their positions and to avoid collisions with vehicles, which can cause them fatal injuries.

The evaluation session lasted one week and took place at our university. The participants were asked to navigate the three maps using an IW and an EPW. They repeated the task 10 times alternating the control modes between the EPW and the IW. The EPW is a pure manual system without any assistance, that is, it did not have the proposed obstacle avoidance system. On the other hand, the IW is a manual system with the proposed obstacle avoidance system, where the users controlled the wheelchair with a joystick according to the paths suggested in our interface (Figure 7). As illustrated in Section 5.2, the proposed IW system provides two schemes to control the wheelchairs: system control and joystick control. Then, to prevent dangerous accidents, we asked the users to manually control the wheelchairs, both the EPW and the IW, by the wheelchair joystick. For the users who were incapable of using the joysticks due to their severe physical impairments, we provided our vision-based interface and explained how to use it. For User V, our vision-based interface was provided. In addition, we asked one caregiver to walk alongside the participants during the entire course of experiments in order to prevent dangerous situations that may arise.

When using a general EPW, users must correctly recognize all surrounding environments and then control the wheelchair to correctly follow their route and to avoid collisions with obstacles as depicted in the respective maps. In contrast, the proposed IW navigation system provides three main functions to users: (1) estimation of the background model under various types of illumination; (2) detection of various obstacles such as the walls as shown in Figure 12a and several obstacles as shown in Figure 12b,c; and (3) correct recommendation of viable paths using the SVM-based classifier. For the third function, users control the wheelchairs according to the paths recommended by the proposed navigation system.

All trials were video recorded and data was collected from the wheelchairs (odometry and readings). During the experiments, we recorded the time taken for the participants to reach each destination and the number of collisions caused by false recognition of environmental information or incorrect wheelchair control.

Then, to compute the trajectories of the participants’ traveling, we implemented two methods and employed them together: one is to use wheelchair motor encoders and the other is to use sensors that are integrated into the smartphone—a gyroscope and an accelerometer.

The former method uses the wheelchair motor encoders that are attached to both wheels of the wheelchair. With wheelchair motor encoders, we can read the number of rotating wheels at the respective motors. Based on them, we can measure wheelchair’s direction and its moving distance, so as to calculate users’ trajectories [31]. This method is simple and widely used in many research works; however, it is likely to be sensitive to systematic or non-systematic errors. To address this problem, we implemented a user trajectory recoding method on a smartphone, and asked the participants to travel with this smartphone during field tests.

Algorithm 1 shows the proposed user trajectory recording method implemented on the smartphone. Using the sensors that are already integrated into smartphones, the paths can be constructed automatically while the user is moving. Here, two inertial sensors are used: a gyroscope and an accelerometer. All paths are recorded in the queue (*Q*) and each path is formatted as one instruction statement, *I*(*A*, *SC*, $\theta_{current},P)$. Thus, the action (*A*) should be defined first among “go straight”, “turn”, and “stop”, and then the related parameters, e.g., step counts (*SC*), compass direction (*θ*), and current position (*P*) should be estimated. In this method, these parameters are calculated based on the sensory information. Using this method, we can record all paths of a user from the starting point to the destination. After finishing each trial, the instruction is pulled out according to the order in which it was recorded, and employed to map the user’s trajectory.

**Algorithm 1.** Proposed user trajectory recoding algorithm.***Input*:** Gyroscope sensor *G*, Accelerometer sensor *AC*, *destination D****Output*:** Stack *S* that contains the set of instructions, *I*(*A*, *SC*, $\theta_{current},P)$, where *A, SC*, θ, *P* are the variables for action, step count, compass direction and position, respectively**Procedure:**1**Initialize**
*A* ← null, $\theta_{previous}$, $\theta_{current}\leftarrow 0{}^{\circ}$, *SC* ← 0, *P*($P_{x}$, $P_{y}$) ← (0,0);2// **Determine the action type among “go-straight”, “turn”, and “stop”** If *AC* < 0.03, then *A* ← *Stop*3else if $|\theta_{current}-\theta_{previous}|>15{}^{\circ}$ then A ← Turn4else *A* ← *Go straight*5**//Estimate the instruction parameters according to the action type** if *A* is *Go straight*, then *SC*, $P_{x}$, $P_{y}$ is updated by the following equation:
$$SC\leftarrow SC+1,\;P_{x}\leftarrow \;SC\cdot \cos\theta_{current},\;P_{y}\leftarrow \;SC\cdot \sin\theta_{current}$$6else if *A* is *Turn*, then $\theta_{current}\leftarrow \;\theta_{previous}$7**Push**
*I*( *A*, *SC*, $\theta_{current},P)$ to *Q*8**//check if the current positioning information is the destination** if the current location is destination, then terminate9else **Go to Line 2**

In this study, in order to obtain more accurate user trajectories, both results obtained from wheelchair motor encoders and smartphone are combined.

After finishing each trial, the participants were interviewed to ascertain their opinion about the proposed IW and the navigation algorithm. The results are discussed in Section 7.4.

### 7.3. Test Results

Here we show the experimental results of the field test that are performed on the five participants. The participants were asked to repeat the task 10 times, alternating the control modes between EPW and IW with and without the proposed navigation system, respectively.

We evaluated the performance in terms of task time and navigation errors: (1) the mean task time was estimated as the time spent to complete the task; (2) navigation errors were measured by the number of collisions with obstacles.

**Figure 13:**
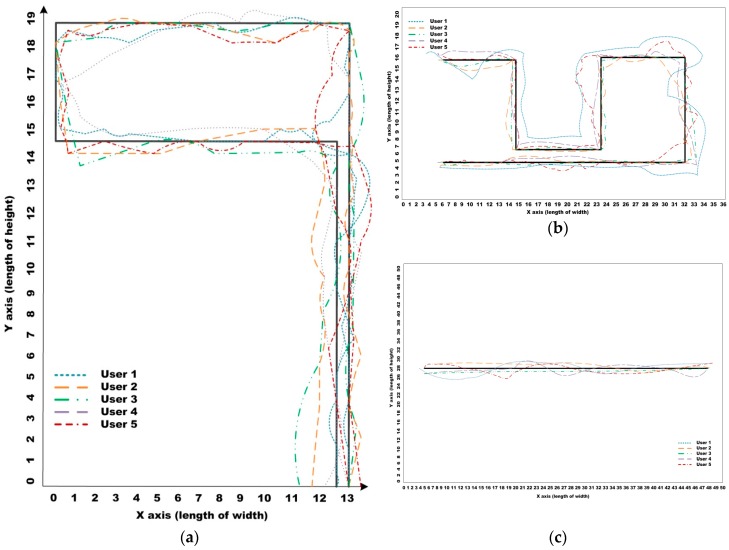
Users’ trajectories in the respective test maps: (**a**–**c**) users’ trajectories in the test maps shown in Figure 12.

Figure 13 shows the accumulated tracks of the five participants made using the proposed IW, each of which was calculated by averaging their tracks for 10 trials. In Figure 13, each colored dashed line denotes one participant’s trajectory, and the optimal path is denoted as an uninterrupted line. The optimal paths were generated by the people with no impairments. Figure 13a,b show the results for the first and second maps in indoor environments, and Figure 13c shows the results for the third map in an outdoor environment.

As mentioned in Section 7.1, User II and User III were better at using the EPW than the other participants because of their driving experience with the joystick. For this reason, they operated close to the optimal path. On the other hand, Users I and IV had difficulties in controlling the EPW because they were cognitively impaired. Thus, their trajectories were significantly outside the optimal paths. User V, the participant with the severe cord injury, could efficiently control the wheelchair using our vision-based interface. At the beginning of the experiments, she was poor at using our IW interface, but she gradually became accustomed to using the interface, and thus her traveling time decreased. Although some differences were discovered among the participants, the participants could successfully travel using the proposed navigation and interface tools. During the experiments, we recorded the total time taken for the participants to reach each destination and the number of collisions caused by incorrect wheelchair control.

Table 10 shows the average task times for the respective participants for the three maps. The lengths of the best routes were 44 m, 119 m and 50 m on the respective maps. According to the user types, different results were generated. Users II and III, who had only motor disabilities, completed the travel more quickly when using a general EPW than the proposed IW, but the difference was not significant. The reason for such a difference is that they had a great deal of experience using an EPW; thus, we expect the traveling time between the two methods to be reduced if users have enough training time to learn the operation of the proposed IW. On the other hand, the proposed IW was more effective for Users I and IV, who had multiple disabilities, such as motor and cognition disabilities, and for User V, who had a severe cord injury. In particular, for the second and third routes, which include a complex background and several obstacles, the proposed IW significantly reduced the traveling time. This result tells us that the proposed method could be more effective for the elderly and the disabled with motor and cognition impairments.

**Figure 14:**
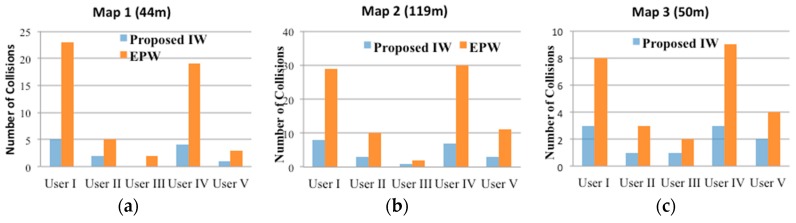
Number of collisions for the respective participants to accomplish the respective goals using a general EPW and the proposed IW navigation algorithm: (**a**) Result for 1st map; (**b**) result for 2nd map; and (**c**) result for 3rd map.

Figure 14 shows the hit ratios of the EPW and IW as participants moved from the starting point to the destination along the three different mapped routes. As can be seen, all users had significantly more collisions when using an EPW than when using an IW: with the general EPW, 8.5 collisions occurred on average. In contrast, the proposed method reduced the number of collisions to 2.0 on average. In particular, the proposed system seemed to be more effective for Users I, IV and V. Users I and IV suffer from cognitive and motor impairments caused by dementia so they could not recognize some dangerous situations. When using the IW, they dramatically reduced the number of collisions with more ease. In case of User V, who had severe physical disabilities, that is, SCI, the number of collisions was proportional to the travelling distance, not to the complexity of the surrounding environments. She had difficulty travelling on the wheelchair due to the spinal cord injury, and thus long distances fatigued her. The main reason for the performance gap between Users I, IV and V was caused by the different schemes that controlled the wheelchair: User V used our vision-based wheelchair interface using face and mouth movements, whereas Users I and IV directly controlled the wheelchair using joystick movement.

Although the proposed method significantly reduced collisions with vehicles or walls, some collisions still occurred. The reason such collisions occurred with the proposed method are as follows: first, even after the correct viable paths were given to the users by the proposed navigation algorithm, they had the difficulties in controlling the wheelchair joystick to stop or change direction in time to prevent collisions; second, the users did not have enough comprehension of the proposed IW size and capability. Due to the attachment of some range sensors, the IW is larger than a general EPW, and this probably caused some collisions when direction was changed by the wheelchair joystick. These problems can be solved in several ways: (1) if the users have enough training time to learn to control the joystick of the IW, the number of collisions can be reduced; (2) if the full autonomous control by our system control is employed or if our vision-based wheelchair interface is provided with the navigation algorithm, the number of collisions can also be decreased. Thus, our future work will include a field test with the proposed IW using full autonomous controls on more participants with more various disabilities, including age, traumas, and injuries.

Our results demonstrated that the IW can support trustworthy safety and prevent accidents that would result in fatal injury to the user. In particular, collisions in outdoor traffic intersections can result in fatal injuries, but these would not occur with the IW. In addition to the IW reducing the number of collisions, it can reduce the time required to complete navigation tasks. Overall, the results of the experiments demonstrated that all participants could complete the navigation mission along the tracks with relative safety and ease.

### 7.4. Post-Test Interview Results

After finishing the field test using the proposed system, the participants were interviewed to investigate their level of satisfaction. To obtain more details about their opinion, the following three questions about the proposed system were asked of the participants, and they answered with a 5-point Likert scale.

■ E1: How helpful was the system when you were moving?
■ E2: How convenient was it to use the system?
■ E3: Do you want to use this system again if it is developed into the general wheelchair?

Most participants were satisfied with the proposed navigation system. The participants responded with average satisfaction rates of 96%, 100%, and 92% for each question, respectively. During the interviews, all participants answered that they experienced less physical damage and required less effort to complete the navigation when using the IW.

## 8. Conclusions

This paper presented a new intelligent wheelchair (IW) navigation system to help disabled or elderly people travel more safely. Using a combination of a camera sensor and eight ultrasonics, the proposed system avoids collision with diverse obstacles, including static obstacles and moving obstacles, such as walking people and moving vehicles, all of which can cause serious harm to people in wheelchairs. The proposed system provides two important functions: (1) obstacle avoidance that detects various obstacles and generates viable paths to avoid collisions; and (2) situation recognition that discriminates the types of outdoor places as sidewalks, roadways or traffic intersections.

For evaluating the coverage of the proposed IW navigation, a total of 80,000 images was collected from various indoor and outdoor areas and the experiments were conducted. The experimental results demonstrated that the proposed navigation algorithms can recognize outdoor situations with 98.3% accuracy and generate avoidable paths with 92.0% accuracy in indoor and outdoor environments. Furthermore, in order to show its practicality, a field test was undertaken with five disabled users, which demonstrated that all the participants could carry out the navigation mission along the circuit with relative safety and ease. In addition, during post-test interviews, all the participants answered that they experienced less physical damage and used less effort in completing the navigation when using the IW.

Future study will proceed on several fronts. To fully show the coverage of the proposed IW system as mobility aids for the disabled and elderly people, field testing must be performed with more participants from a more various population, such as people who are aging or suffering from trauma or brain injury. We are currently working on this issue as well as improving each component.

## Figures and Tables

**Table 1:** The decision function in order to determine the safe paths.

Input: real time image streaming *I*, 8 sensors values *S*
Output: viable paths v = {go, stop, turn-left, turn-right}

Selecting the viable paths on the converter (*I*, *S*)
{
*a* = Situation awareness (*I*);
*b* = Sensor-based obstacle avoidance (*S*);
*c* = Vision-based obstacle avoidance (*I*);

if (*a*== ‘intersection’) *v* ←‘stop’
else if (*b*==‘stop’) *v* ←‘stop’
else *v* ←*c*;

Interface (*v*);
}

**Table 2:** Operation voltages of the proposed IW system.

Directions	Output 1 (Right Motor)	Output 2 (Left Motor)
Go straight	2.45 V	2.45 V~3.7 V
Stop	2.45 V	2.45 V
Turn left	1.2 V~3.7 V	2.45 V
Turn right	2.45 V~3.7 V	2.45 V
Back	2.45 V	1.2 V~3.7 V

**Table 3:** Data collections used to evaluate the proposed obstacle avoidance method.

Places	DB Sets	Illumination	Background Texture	Obstacles
Indoor	DB I Daytime	- Fluorescent lighting and partial sunlight	- Little reflection - Weakly textured floor	- Only static obstacles
	DB II Daytime	- Pin lighting and partial sunlight	- High reflection - Marble textured or highly textured floors	- Static and dynamic obstacles
	DB III Nighttime	- Fixed illumination with pin light	- High reflection - Marble textured or highly textured floors	- Static and dynamic obstacles
Outdoor	DB IV Daytime	- Direct sunlight with little shadow	- Weakly textured ground with small road signs	- Static obstacles
	DB V Daytime	- Direct sunlight with complex shadow	- Reflection by sunlight - Highly textured ground	- Static and dynamic obstacles (moving people and vehicles)
	DB V Nighttime	- Soft glow of street lamp	- Highly textured ground	- Static and dynamic obstacles

**Table 4:** Average performance of the three methods (%).

Methods	SVM-Based Method	NN-Based Method	VFH-Based Method
Indoor	88.0	83.8	68.0
Outdoor	92.0	89.0	68.8
Average	90.0	86.4	68.4

**Table 5:** Average time to be taken to a frame in obstacle avoidance module (ms).

Methods	VFH-Based Method	NN-Based Method	SVM-Based Method
OGM Generation	296	2.95	2.95
Path Recommendation	0.45	0.77	2.08
Total	296.45	3.27	5.03

**Table 6:** Data collection used to evaluate the situation recognition method.

Environmental Factors		The Number of Images	Background Texture	Obstacles
Illumination Condition	Scene Types			
Direct sunlight with little shadow	Ground with highly textured patterns, some static obstacles	110	Dataset 1	Training data
	Textured ground, some moving obstacles (people, vehicle)	64	Dataset 2	
	Ground with highly textured patterns, simple outdoor structures	844	Dataset 3	Test data
	Textured ground with static obstacles	256	Dataset 4	
Direct sunlight with complex shadow	Simple ground with no specific patterns, simple outdoor structures	156	Dataset 5	
	Ground with textured patterns, complex outdoor structure	312	Dataset 6	

**Table 7:** Overall performance of the proposed situation recognition method (%).

	Dataset 1	Dataset 2	Dataset 3	Dataset 4	Dataset 5	Dataset 6	Total
Accuracy	91.0	95.3	100	100	94.8	97.4	96.42

**Table 8:** Average time to be taken to a frame in situation recognition module (ms).

Modules		Processing Time
Situation Recognition	Preprocessing	41.24
	Texture classification	181.32
	Shape filtering	5.98
Total		228.54

**Table 9:** Participants’ profiles.

	Age/Gender	Experience in Using EPW	Type of Disabilities	
			Motor	Cognition
**User I**	60-year-old male	Yes	Lower body amputation by diabetes	Low cognition/reflexes by dementia
**User II**	48-year-old female	Yes	Amyotrophic lateral sclerosis	-
**User III**	32-year-old female	Yes	Cerebral palsy	-
**User IV**	73-year-old male	Yes	Lack of strength	Weak senile dementia
**User V**	28-year-old female	Yes	Spinal cord injury	-

**Table 10:** Average time taken for participants to reach the three destinations.

	Map 1 (44 m)			Map 2 (119 m)			Map 3 (50 m)		
	Proposed IW	EPW	Difference	Proposed IW	EPW	Difference	Proposed IW	EPW	Difference
User I	158	264	−106	315	419	−104	112	214	−102
User II	114	95	+19	164	152	+12	91	81	+10
User III	115	97	+18	170	145	+25	83	62	+21
User IV	284	320	−36	485	594	−109	254	294	−40
User V	131	296	−165	281	420	−139	94	380	−286
Average	160.4	214.4	−54	283	346	−63	126.8	206.2	−79.4
Standard deviation	71.3	109.8	38.5	131.1	193.9	75.7	71.8	136.4	64.6
